# Establishing the Secondary Metabolite Profile of the Marine Fungus: *Tolypocladium geodes* sp. MF458 and Subsequent Optimisation of Bioactive Secondary Metabolite Production

**DOI:** 10.3390/md15040084

**Published:** 2017-03-23

**Authors:** Bethlehem Kebede, Stephen K. Wrigley, Anjali Prashar, Janina Rahlff, Markus Wolf, Jeanette Reinshagen, Philip Gribbon, Johannes F. Imhoff, Johanna Silber, Antje Labes, Bernhard Ellinger

**Affiliations:** 1 Hypha Discovery Ltd., Russell Building, Brunel Science Park, Kingston Lane, Uxbridge, Middlesex UB8 3PQ, UK; bethlehem.kebede@gmail.com (B.K.); stephen.wrigley@hyphadiscovery.co.uk (S.K.W.); dr_anjaliprashar@hotmail.com (A.P.); 2 Fraunhofer IME ScreeningPort, Schnackenburgallee 114, 22525 Hamburg, Germany; janina.rahlff@uni-oldenburg.de (J.R.); markus.wolf@ime.fraunhofer.de (M.W.); jeanette.reinshagen@ime.fraunhofer.de (J.R.); philip.gribbon@ime.fraunhofer.de (P.G.); 3 Helmholtz Centre for Ocean Research (GEOMAR), Am Kiel-Kanal 44, 24106 Kiel, Germany; jimhoff@geomar.de (J.F.I.); jsilber@geomar.de (J.S.); 4 Flensburg University of Applied Sciences, Kanzleistr. 91–93, 24943 Flensburg, Germany; antje.labes@hs-flensburg.de

**Keywords:** *Tolypocladium geodes*, tolypocladenol C, NCI60, malettinin B, malettinin E, fermentation development, activity guided purification, anticancer

## Abstract

As part of an international research project, the marine fungal strain collection of the Helmholtz Centre for Ocean Research (GEOMAR) research centre was analysed for secondary metabolite profiles associated with anticancer activity. Strain MF458 was identified as *Tolypocladium geodes*, by internal transcribed spacer region (ITS) sequence similarity and its natural product production profile. By using five different media in two conditions and two time points, we were able to identify eight natural products produced by MF458. As well as cyclosporin A (**1**), efrapeptin D (**2**), pyridoxatin (**3**), terricolin A (**4**), malettinins B and E (**5** and **6**), and tolypocladenols A1/A2 (**8**), we identified a new secondary metabolite which we termed tolypocladenol C (**7**). All compounds were analysed for their anticancer potential using a selection of the NCI60 cancer cell line panel, with malettinins B and E (**5** and **6**) being the most promising candidates. In order to obtain sufficient quantities of these compounds to start preclinical development, their production was transferred from a static flask culture to a stirred tank reactor, and fermentation medium development resulted in a nearly eight-fold increase in compound production. The strain MF458 is therefore a producer of a number of interesting and new secondary metabolites and their production levels can be readily improved to achieve higher yields.

## 1. Introduction

The urgent need for novel substances for the treatment of severe human diseases such as cancer, combined with the recognition that marine organisms provide a rich potential source of such substances, support the intensive exploration of new substances from marine organisms [1]. Oceans are sources of a large group of structurally unique natural products that are mainly accumulated in marine macroorganisms such as invertebrates (e.g., sponges, soft corals, tunicates) and algae. Several of these secondary metabolites have pronounced pharmacological activities [2]. Microorganisms such as fungi and bacteria are truly prolific producers of bioactive molecules and an increasing number of studies support the hypothesis that many compounds originally thought to be produced by macroorganisms actually come from associated microbes [3,4,5]. To adapt to, and survive in, the marine ecosystem, characterized by very special conditions that differ from those found in other habitats, marine microorganisms sometimes produce structurally unique bioactive secondary metabolites not found in terrestrial organisms [6]. 

Although intensive research is necessary to unravel the important resources of the ocean, marine biotechnology has now started to develop integrated strategies. During past decades, one of the most serious bottlenecks in developing natural products from marine sources has been the availability of biomass and/or of optimised cultivation conditions to gain sufficient amounts of substances for preclinical and clinical studies. The concentrations of the compounds of interest are often minute, sometimes accounting for less than 10^−6^ percent of the wet weight of the macroorganism [7]. Exploitation is further complicated by the fact that most of these metabolites possess highly complex structures, making an economical production via chemical synthesis difficult. Many unsuccessful attempts have been made to extract these substances in sufficient amounts from invertebrates and algae including harvesting from appropriate sites, aquaculture and even cell culture of the respective organisms [8]. Prominent examples are the bryostatins, the halichondrins and other antitumoral or anti-inflammatory active substances from marine invertebrates. In these cases, the content in the animals was very low (less than 1 g per ton of biomass) and it was not possible to harvest such large amounts of organisms from nature without destroying their habitats, nor was it possible to cultivate the organisms or cell cultures thereof in sufficient scale and appropriate time [9]. 

In contrast, the focus on microbes associated with marine macroorganisms is a highly sustainable approach aiming to conserve natural habitats, since only small pieces of tissue of macroorganisms are required. These microbes are considered to be an excellent source of secondary metabolites involved in the interspecies communication because they presumably evolved for specific functions such as protecting the host and/or the producer against competitors and/or diseases. From a biotechnological point of view, many of these compounds have pharmaceutical properties, often antibiotic or cytotoxic, that may be useful as lead structures for the development of new drugs [10]. Therefore, the use of marine fungi, which can easily be grown in the laboratory and at large scale, and that are prolific producers of bioactive compounds, including anti-tumoral substances, provides a solution for the supply issue. 

In all cases studied, marine fungi have been revealed to produce natural products (NP) for a variety of ecological purposes. Fungal natural products are often produced in response to a plethora of environmental cues, which might be also small molecules [11]. Despite their potential for secondary metabolite production, marine fungi are still poorly characterised and underutilised for biotechnological application [12]. A literature survey covering more than 23,000 bioactive microbial products, i.e., antifungal, antibacterial, antiviral, cytotoxic and immunosuppressive agents, shows that the producing organisms are mainly from the fungal kingdom. Hence, fungi represent one of the most promising sources of bioactive compounds [13,14]. Prominent examples of compounds isolated from marine fungi comprise ulocladol, halimide, avrainvillamide, pestalone, and the halovirs A-E [15,16,17]. However, the number of available strains from marine sources is limited and the knowledge of marine fungi in general is scarce. For example, there is a deficit in systematic research of the potential of marine fungi in different application fields and the availability of new structures of bioactive compounds, especially targeted against cancer, is still low [18].

Within the comprehensive screening approach of the EU FP7 project MARINE FUNGI, the potential of secondary metabolites from fungi associated with marine macroorganisms was evaluated to provide lead compounds for the development of cancer treatments. Extracts from fermentations of fungi isolated from Mediterranean sponges, Indonesian corals and Chilean macroalgae were screened against tumour cell lines [19]. 

Secondary metabolite biosynthesis has long been known to depend on environmental cues, including carbon and nitrogen sources, ambient temperature, light and pH [20]. As a consequence, changes to culture conditions can largely modify the spectrum and amounts of secondary metabolites produced by fungi [21]. A number of stimuli, such as culture media and cultivation condition changes, which are known to induce the production of secondary metabolites [22], were employed to induce variation of the metabolite pattern of the isolated marine fungi. 

In our investigation, extracts from fermentations of the sponge-associated strain MF458, subsequently identified as *Tolypocladium geodes*, were found to have potent anti-tumour effects derived predominantly from anti-proliferative rather than overtly cytotoxic profiles. As small-scale assay-guided fractionation did not reveal the obvious presence of compounds known to have anti-tumour properties, we commissioned larger scale fermentations. Extracts of these fermentations were rich in diverse secondary metabolites. The strain was cultivated in a variety of media in order to characterise the metabolite spectrum of this strain and its susceptibility to stimulation by changing cultivation conditions [21]. 

*Tolypocladium* spp. have attracted significant attention as producers of bioactive secondary metabolites. Efrapeptins, pyridoxatin and terricolin have previously been reported as products of terrestrial isolates of *T. geodes*, while production of the medicinally-significant cyclosporins is usually associated with other *Tolypocladium* spp. [23]. Metabolites from marine *Tolypocladium* spp., such as the new efrapeptin J, have also been reported [24].

## 2. Results

### 2.1. Classification of Strain MF458

**Figure 1:**
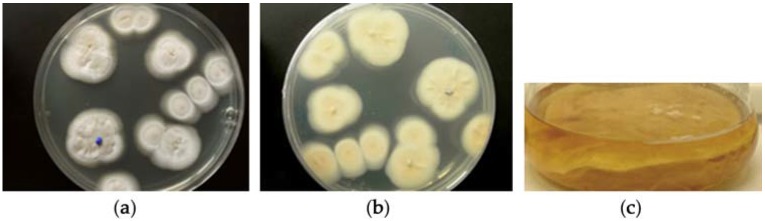
MF458; agar colony on WSP30 medium after 21 days of incubation at 22 °C. (**a**) Image taken from above the plate; (**b**) Image taken from below the plate; (**c**) a liquid culture (non-shaken) resulted in white mycelium growing mainly subsurface.

The fungal strain MF458 is part of the marine fungal strain collection of GEOMAR. On Wickerham agar, the strain grew in whitish colonies (Figure 1), and produced *Acremonium*-like branched conidophores. The sequences of the ITS rRNA genes comprised 501 nucleotides, which exhibited a similarity of 93% for the ITS gene sequence of a *Tolypocladium geodes*. The 18S RNA gene sequence was also obtained, comprising 1827 nucleotides, and showed the highest degree of similarity (93%) to a *Cordyceps pleuricapitata*.

### 2.2. Isolation and Identification of ***1**–**6***

**Figure 2:**
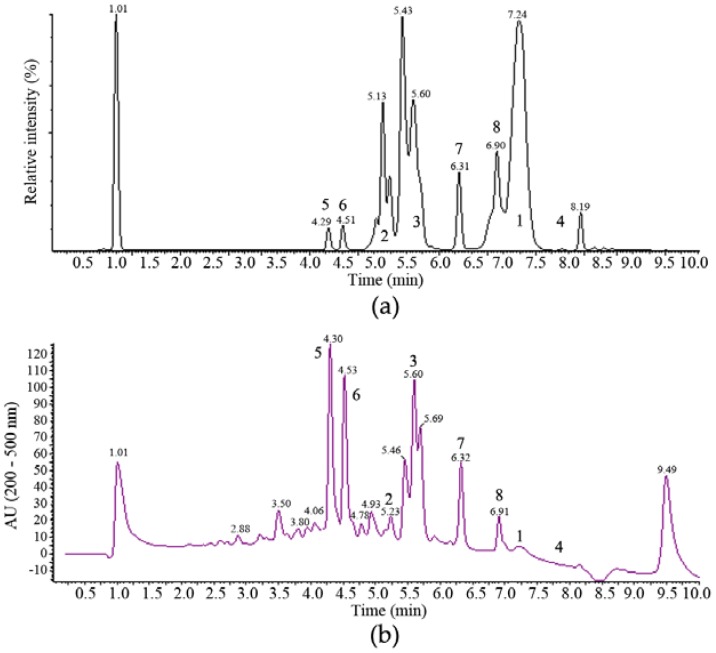
Reversed phase HPLC chromatogram of the whole culture ethyl acetate extract of a 10 L MF458 fermentation analysed on a Symmetry Shield RP8 column with gradient elution (**a**) evaporative light scattering detector chromatogram; (**b**) diode array UV-visible (200–500 nm) chromatogram.

**Figure A1:**
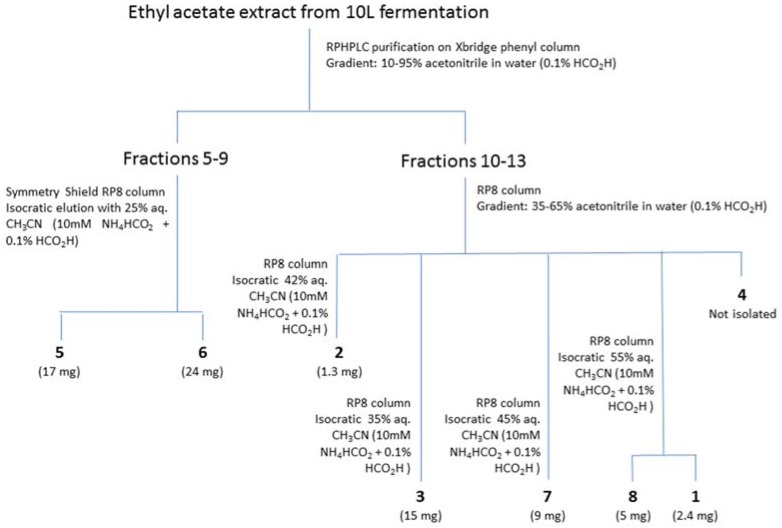
Scheme of the developed purification methods to obtain compounds **1** to **8**.

The anti-tumour activity of the ethyl acetate extracts of fermentations of MF458 were associated with multiple chromatographic peaks that were separable by reversed phase HPLC (Figure 2) and purification methods were developed accordingly (summarised in Appendix A Figure A1).

One major band of activity was associated with lipophilic components eluting from towards the end of the reversed phase gradient employed for the separation illustrated in Figure 2a (retention time 7.0 to 7.5 min). These components had very little UV absorbance and ESI-MS indicated molecular weights of 1188, 1202, 1216 and 1218. The major component from this band with a molecular weight of 1202 ([MH]^+^ at *m*/*z* 1203, [MNH_4_]^+^ at *m*/*z* 1220, [M − H]^−^ at *m*/*z* 1201, [M + formate]^−^ at *m*/*z* 1247) was purified and its ^1^H NMR spectrum in CDCl_3_ was identical with that of the known *Tolypocladium* spp. metabolite cyclosporin A, **1** [25]. 

A more polar band of activity eluting from 5.0 to 5.5 min (Figure 2a) was also associated with components of relatively high molecular weights. Again, these compounds had no significant UV absorbance and molecular ions were detected in ESI^+^-MS at *m*/*z* 1607, 1621, 1635, 1649 and 1663, which are consistent with the presence of the known *Tolypocladium* spp. metabolites efrapeptins, being positively charged linear peptides [26].

The major component of this band was isolated and had a molecular weight of 1621. Its ^1^H NMR spectrum in CDCl_3_ was consistent with that expected for efrapeptin D, **2**. Another major active metabolite produced in the fermentation eluted just after the efrapeptins with a retention time of 5.6–5.7 min (Figure 2b). This had a UV spectrum with maxima at 218 and 289 nm and ESI-MS indicated a molecular weight of 263 ([MH]^+^ at *m*/*z* 264 and [M − H]^−^ at *m*/*z* 262). Its ^1^H and ^13^C NMR spectra were identical with those reported for pyridoxatin, **3**, first reported as an *Acremonium* sp. metabolite [27]. Interestingly, a late-eluting fraction in the chromatogram was found to be related to pyridoxatin. Its ESI-MS indicated a molecular weight of 842 ([MH]^+^ at *m*/*z* 843.6 and [M − H]^−^ at *m*/*z* 841.7) and its UV spectrum consisted of maxima at 221, 246, 287 and 388 nm. These characteristics match well with those reported for terricolin, **4**, a (pyridoxatin)_3_Fe complex reported as a product of *Tolypocladium terricola* [28].

Two more polar compounds with significant anti-tumour activity (eluting with retention times of 4.3 and 4.5 min in Figure 2) were obviously closely related with the same molecular weight of 292 (deduced from ESI-MS molecular ions at *m*/*z* 293 ([MH]^+^) and 291 ([M − H]^−^) in positive and negative ionisation modes, respectively) and identical UV spectra with maxima at 253, 328_sh_ and 359 nm. This UV spectrum is characteristic of the presence of a tropolone moiety and these compounds were identified by their ^1^H and ^13^C NMR spectra. The earlier eluting material was found to be identical to the fungal metabolite malettinin B, **5**, [29] while the second peak was found to correspond to the recently described malettinin E, **6** [30], and its identity was confirmed by a direct NMR comparison with authentic material of this compound. These malettinins have not previously been described as *Tolypocladium* spp. metabolites. A polyketide origin has been proposed for the malettinins, with the tropolone unit perhaps arising via an aromatic ring expansion, as may be the case for other fungal tropolones [31].

### 2.3. Structure Elucidation of ***7*** and ***8***

Compounds **7** and **8** are represented by the peaks eluting at 6.3 and 6.9 min in Figure 2. The physico-chemical properties, ^1^H and ^13^C NMR spectral data for compound **7** are summarized in the Appendix A Table A1 and Table A2, respectively. The ESI-MS spectrum of compound **7** gave a molecular ion ([MH]^+^) at *m*/*z* 358, indicating a molecular weight of 357, and accurate mass measurement on this ion was consistent with the molecular formula C_21_H_27_NO_5_ (Appendix A Table A1). The ^1^H and ^13^C NMR data for 7 are summarized in Appendix A Table A2. The ^13^C NMR spectrum confirmed the presence of 21 carbon atoms and DEPT spectra indicated that these comprised 3 methyl, 3 methylene, 9 methine and 6 quaternary carbons. The 3 protons unaccounted for were not observed in the ^1^H spectrum in CD_3_OD and were inferred to be exchangeable. The presence of a *p*-substituted phenyl moiety was evident from the observation of four sp2 methine carbons as two coupled pairs of chemically equivalent protons C-2′/C-6′ (131.8/6.99 ppm) and C-3′/C-5′ (116.0/6.65 ppm). The presence of a tetramic acid moiety was suggested by broad carbon signals at 198.2 (C-4), 197.3 (C-7), 176.7 (C-2), 103.0 (C-3) and 63.0 (C-5) ppm. ^1^H coupling between methine H-5 at 3.95 ppm and methylene protons H-6 at 2.98 and 2.82 ppm together with long range ^1^H-^13^C correlations observed in the HMBC spectrum between the methylene group at C-6 (37.9/2.98, 2.82 ppm) and carbon signals at C-4 (198.2 ppm), C-1′ (128.1 ppm) and C-2′/C-5′ (131.8 ppm) indicated that this methylene bridged between the *p*-substituted phenol and tetramic acid moieties. This conclusion was also consistent with the UV maxima observed for **7** (Appendix A Table A1). COSY, HSQC and HMBC data were used to establish the presence of a 5,7-dimethylheptan-2-enyl side chain. HMBC correlations between the H_3_-16 methyl protons at 1.01 ppm and H_2_-9 methylene protons at 1.72/1.04 ppm, and C-7 at 197.3 ppm indicated the point of attachment of this side chain to the tetramic acid ring to establish the structure of **7** as 5-(4-hydroxybenzyl)-3-(2,4-dimethyl-1-hydroxyoct-6-en-1-ylidene)pyrrolidine-2,4-dione. Due to its structural similarity to the tolypocladenols from another *Tolypocladium* species, *T. cylindrosporum*, we propose to assign this molecule to this family and name it tolypocladenol C. 

**Figure 3:**
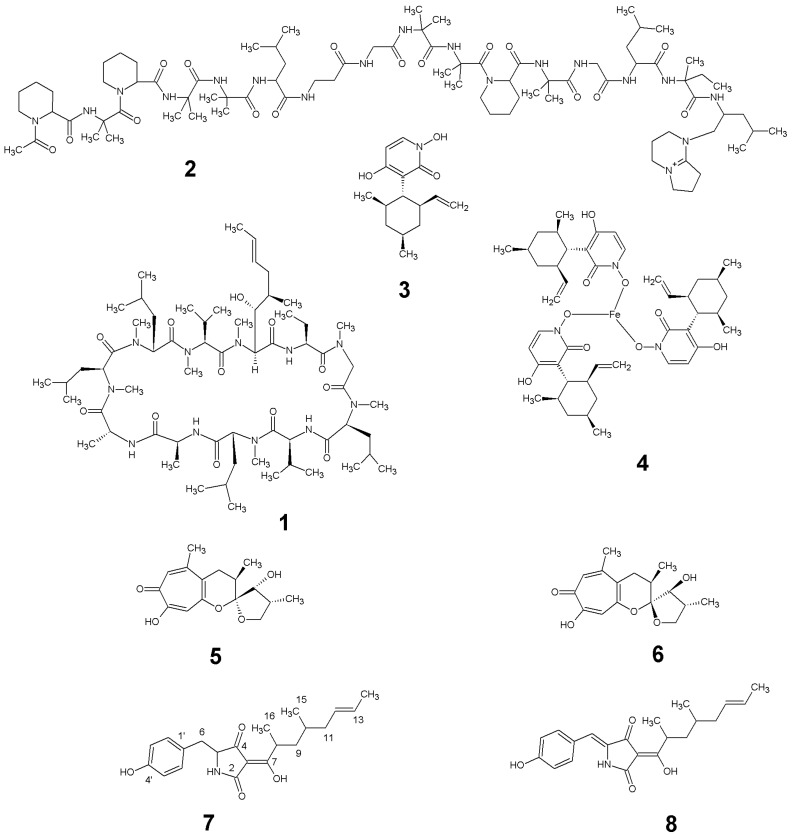
Natural products produced by *Tolypocladium geodes* sp. MF458: cyclosporin A (**1**); efrapeptin D (**2**); pyridoxatin (**3**); terricolin (**4**); malletenin B (**5**); malletenin E (**6**); tolypocladenol C (**7**); tolypocladenols A_1_ and A_2_ (**8**).

The ESI-MS spectrum of compound **8** gave a molecular ion ([MH]^+^) at *m*/*z* 356, indicating a molecular weight of 355, and accurate mass measurement on this ion was consistent with the molecular formula C_21_H_25_NO_5_. It had a more extended UV spectrum than **7**, indicating a greater degree of conjugation. Its NMR spectra indicated a close structural relationship to **7** and interpretation of the NMR data indicated that **8** is the 5,6-dehydro derivative of **7**: 5-(4-hydroxybenzylidene)-3-(2,4-dimethyl-1-hydroxyoct-6-en-1-ylidene)pyrrolidine-2,4-dione. After this work was completed, compound **8** was reported as tolypocladenols A_1_/A_2_, isolated from fermentations of the endolichenic fungus *Tolypocladium cylindrosporum* [32]. The NMR, UV and MS data for 8 are consistent with those reported for tolypocladenols A_1_/A_2_ and are not reported herein. Tolypocladenols A_1_/A_2_ were identified as a mixture of enantiomers with *RR* and *SS* configurations at carbons 8 and 10 and their structures were determined by single crystal X-ray diffraction. In addition to the tolpocladenols A1, A2 and B, **7** and **8** are structurally similar to a number of other fungal acyltetramic acids described in the literature. Examples would be militarinones B and C [33] and epicoccarines A and B [34], with **7**, showing a high degree of structural similarity to the epicoccarines, with their mostly saturated acyl chains. Due to the structural similarity between compound **8** and **7** it can be assumed that the biosynthesis of both natural products is analogous to the hybrid PKS-NRPS pathway proposed for a variety of tetramic acid based fungal natural products, such as the epicoccarines A and B [32,35]. Figure 3 summarises the natural products isolated from *Tolypocladium geodes* MF458.

### 2.4. Biological Activities of ***1**–**8***

**Figure 4:**
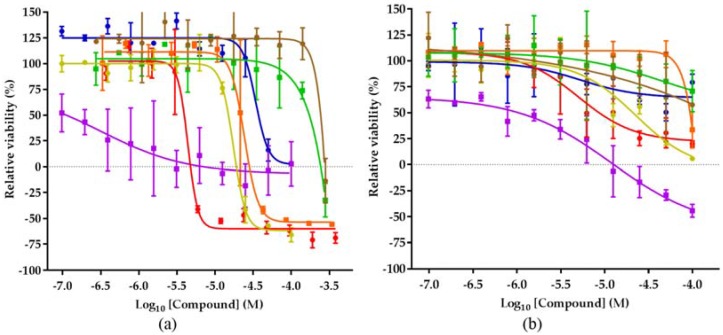
Activity of the isolated compounds in different cell lines. (**a**) Viability of MCF-7 cell line after 48 h compound treatment. (**b**) Viability of OVCAR-5 cell line after 48 h compound treatment; cyclosporin A (blue), efrapeptin (violet), malettinin B (orange), pyridoxatin (red), malettinin E (dark yellow), tolypocladenol C (green), tolypocladenol A_1_/A_2_ (brown).

The biological activity of semi-purified chromatography fractions was initially determined using three sensitive cell lines at three concentrations for activity guided purification. The detailed procedure was reported elsewhere [19] and showed a promising activity profile for a number of fractions from MF458. Purified compounds were analysed using a panel of cell lines from the NCI60 panel, consisting of cell lines from 8 different tissues and a number of different cancer stages and genetic characteristics. Terricolin (**4**), as an iron complex, was deprioritised due to unfavourable chemical properties and not tested further. While no general trend with respect to tissue sensitivity could be identified, clear differences between individual cell lines were apparent (see Appendix A Table A3 for complete overview of inhibition values). The growth of the breast adenocarcinoma cell line MCF-7 was inhibited at least at the highest concentration by all the natural products evaluated, while the growth of the ovarian carcinoma cell line OVCAR-5 was reduced to at least 50% only by four of the seven compounds (Figure 4).

The only compound with a similar efficiency in both cell lines is efrapeptin D (**2**), inhibiting the growth of MCF-7 and OVCAR-5 at 7 µM and 12 µM, respectively. The effect of efrapeptin, allosteric inhibition of the mitochondrial ATP synthase, explains its effectiveness in both cell lines and also the broad activity profile of this predominantly cytostatic compound [36]. The malettinins B and E (**5** and **6**) are less studied and little is known about their biological effects [29,37]. This study represents therefore the first description of their cytotoxic potential. The compounds show an activity in every cell line evaluated, except melanoma cell line SK-MEL-28, which is not growth inhibited by malettinin B. Tolypocladenol C (**7**) and also tolypocladenols A_1_/A_2_ (**8**), although less studied, appear to be not active against the majority of cancer cell lines, reducing the relevance of these compounds in terms of anticancer drug discovery. In line with previous reports, pyridoxatin (**3**) was identified as showing a good toxicity profile against the cell line panel in the submicromolar and low micromolar range.

### 2.5. Secondary Metabolite Profile of MF458 Depending on Fermentation Conditions

**Figure 5:**
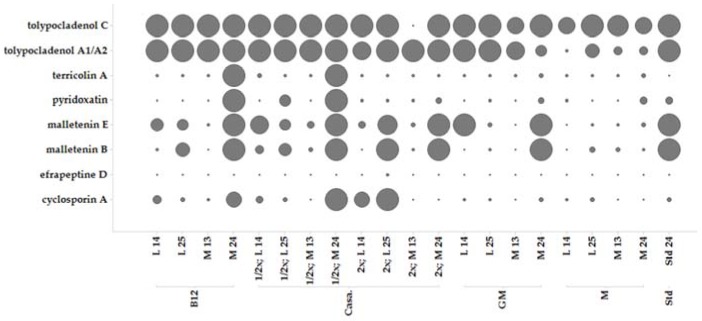
Comparison of production of MF458 natural product profiles in different media in Erlenmeyer (EM) flasks. Peak area (ion count) is given as size and ranges from 0 to 10 M; values above 10 M are shown as maximum; B12: Casamino acid supplemented with B12, Casa: casamino acid medium, half concentrated (½), double concentrated (2×), GM: casamino acid medium containing glucose and malt, M: casamino acid medium supplemented with molasses, L, liquid shaken culture, M. mycelium from standing culture, numbers indicate days of cultivation.

Production of secondary metabolite extracts for screening and leading to the identification and dereplication of the compounds **1**–**8** was achieved by fermentation in Erlenmeyer (EM) flasks at 1 L scale under different conditions (Figure 5). Pre-experiments (not shown) indicated that casamino acids medium seems to be best for production of secondary metabolites by MF458. Therefore, the casamino acids medium recipe was taken as a basis and adopted for optimisation of metabolite production. Production of the metabolites depended on both the medium composition and the shaking conditions.

Most of the compounds were produced best using standing conditions that allowed formation of a mycelium cake. The conditions were used to produce higher amounts of the compounds for purification and isolation purposes. Generally, cultivation had to be performed for >24 days (>550 h) for obtaining maximum yield of the metabolites.

### 2.6. Optimisation of Production for Scaling-Up the Production of the Malettinins

**Figure 6:**
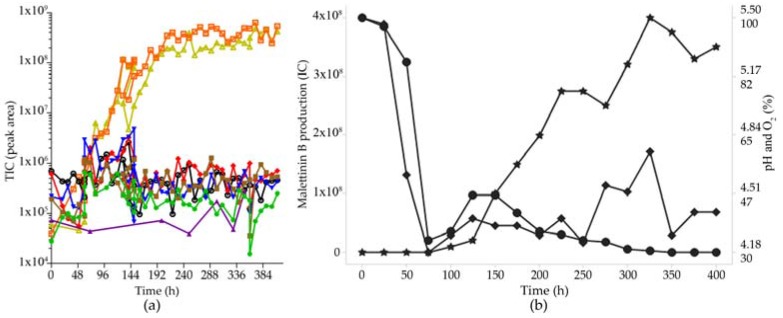
Optimisation of malettinin production. (**a**) Production of secondary metabolites in casamino acid medium supplemented with crystalline cellulose under STR conditions as peak area [total ion count (TIC)]; The color coding is: cyclosporin A (**1**, blue), efrapeptin (**2**, violet), malettinin B (**5**, orange), pyridoxatin (**3**, red), malettinin E (**6**, dark yellow), tolypocladenol C (**7**, green), tolypocladenol A_1_/A_2_ (**8**, brown). (**b**) Production of malettinin B (**5**): amount of malettinin B production (star); pH (ranging from 5.50 to 4.18, shown as point); O_2_ saturation in % (diamond).

For a larger scale fermentation of malettinin E (**6**) and B (**5**) in bioreactors, the production had to be transferred initially into liquid, shaken conditions, as the highest production in the initial screening study was observed in standing cultures. Accordingly, casamino acids medium was supplemented with substrates supporting stable mycelium formation under liquid shaken/stirred conditions, such as crystalline cellulose, paper, gelatine, CaCO_3_, and agar. These pellet enhancers had different effects on the different metabolites, e.g., the addition of CaCO_3_ increased especially the production of tolypocladenol C (**7**) by a factor of 10. As the malettinins had the most interesting biological activity, the optimisation strategy was, however, focused on optimal production conditions for the malettinins. With respect to the pellet enhancers, addition of 10 g/L crystalline cellulose had the best effect on malettinin production. Accordingly, casamino acids medium supplemented with crystalline cellulose was chosen for a stirred tank reactor (STR) cultivation. STR cultures were conducted at the 10 L scale, controlling gas flow and stirrer speed but with an autonomous pH regime. Malettinin production under STR conditions out-competed the production of the other metabolites (Figure 6).

A second STR fermentation was performed with pH control (at 4.3). The total production of the malettinins was lower and the production of the malettinins started 60 h later compared to the STR without pH control. Total yield was confirmed with respect to malettinin B (Table 1).

## 3. Discussion

### 3.1. Phylogenetic Position

The phylogenetic position of the polyphyletic groups *Cordyceps* and *Tolyplocladium* within the *Ophiocordycipitaceae* has been radically reorganised during the last years. Some *Tolyplocladium* sp. have been found to be anamorphs of *Cordyceps* spp. [38], however, based on an exhaustive phylogenetic reconstruction, six genera were newly organised within this taxon including *Tolypocladium* [39]. Accordingly, the genus *Tolypocladium* W. Gams [40] was proposed for protection over the other two generic names in the clade, *Elaphocordyceps* and *Chaunopycnis*. The clade itself is well supported in this and other published analyses [20,41] and the MF458 18S rRNA and ITS sequences do cluster within this clade. Following the one-fungus-one name rule and the recent nomenclatural proposals, our strain MF458 was assigned to the genus *Tolypocladium*, species *geodes* [42]. However, the molecular identity is comparably low and therefore additional parameters must be taken into account. The chemical profile of MF458 supports the molecular classification of the strain into the genus *Tolypocladium*. Despite the limits of using secondary metabolites in phylogeny due to inconsistent distribution throughout the fungal kingdom [43], secondary metabolites can support taxonomic classification and identification: many of the compounds isolated from fermentations of MF458 have previously been reported as *Tolypocladium* spp. metabolites. The efrapeptins [44], pyridoxatin [44] and terricolin [28] have previously been reported as products of terrestrial isolates of *T. geodes*, while production of cyclosporins is usually associated with other *Tolypocladium* spp. [45]. At the time this work was done, compounds **5**–**8** belonged to chemical families that had not been associated with *Tolypocladium* spp. After this work was completed, however, compound **8** was reported as tolypocladenols A_1_ and A_2_, isolated from fermentations of the endolichenic fungus *Tolypocladium cylindrosporum* [32]. Accordingly, the chemical profile of MF458 supports the molecular classification of the strain into the genus *Tolypocladium*. 

### 3.2. Secondary Metabolite Profile

The secondary metabolite profile indicates the expression of at least five different biosynthetic pathways in parallel, since other, unrelated compounds were present in the extracts but only those that had some degree of anti-tumour activity were purified and characterised. The full genome sequence of a related species, *T. inflatum*, revealed 14 NRPSs, 20 PKSs, 4 hybrid PKS/NRPSs, 11 putative NRPS-like enzymes, 5 putative PKS-like enzymes, and one dimethylallyl-tryptophan synthase (DMATS), indicating that *Tolypocladium* spp. in general are prolific secondary metabolite producers [45]. However, no data is available on the parallel operation of these biosynthesis routes. The compounds characterised in this study indicate the co-action of PKS (malettinins B and E), NRPS (cyclosporin A and efrapeptins) and hybrid PKS-NRPS (tolypocladenols and pyridoxatin) pathways.

Secondary metabolite biosynthesis has long been known to depend on environmental cues and as a consequence, changes of culture conditions can largely modify the spectrum and amounts of secondary metabolites produced [21]. *Tolypocladium geodes* MF 458 showed a wide spectrum of metabolites and reacted sensitively to changes of the culture conditions.

### 3.3. Biological Activity Profile

The eight identified natural products produced by MF458 show a diverse spectrum of activities. Cyclosporin A (**1**) is a well-known inhibitor of NFAT signalling and has been used since the 70s as an immunosuppressant in the clinic [46]. Efrapeptin D (**2**) binds to ATP synthase, also inhibiting the HSP90 chaperone function and the proteasome. These multiple targets prevent the development of efrapeptin into a drug although the molecule is effective against MCF-7 induced breast cancer [47]. The malettinins B and E (**5** and **6**) are less studied and little can be found about their biological activity other than that they exhibit weak antimicrobial activity [29,37]. However, their activity against all cell lines evaluated and their generally low cytotoxicity make them interesting molecules for future target deconvolution strategies. Pyridoxatin (**3**) is similarly lacking a conclusive mode of action [48]. It is a compound with previously reported cytotoxicity against various cell lines and moderate inhibitory activity against gelatinase A, a matrix metalloprotease, but also lacking a conclusive cellular mode of action [48]. Instead pyridoxatin was shown to induce erythropoietin gene expression, although a more general effect on gene expression cannot be ruled out since it downregulated genes relevant for ergosterol biosynthesis in *Candida albicans*, as well [49,50]. The compound is reported to be a free radical scavenger, but it remains to be investigated whether this mode of action can explain its activity profile [27]. Tolypocladenol C (**7**) and tolypocladenols A_1_/A_2_ (**8**) appear to be non-cytotoxic and terricolin (**4**) is an iron complex making it also unfavourable for further activity screening.

### 3.4. Fermentation Optimization

Lead optimisation and preclinical development for newly identified natural products is crucially dependent on sufficient supply of material. For this purpose, chemical synthesis and biotechnological production have to be considered and compared in terms of feasibility, effort and costs. However, upscaling of the biotechnological production from, in most cases, Erlenmeyer flask cultures to controllable stirred tank reactors is a challenge, as the scaling itself is a change of cultivation conditions that may lead to a change of the secondary metabolite profile [21]. For MF458, pellet enhancers were used to overcome the initial standing cultivation conditions and to expedite the transfer into STR. The phenomenon of production being restricted to cultures that grew on the surface of the culture broth or at least under adherent conditions is well known for filamentous fungi. For instance, the sorbicillactone A produced by a marine *Penicillium chrysogenum* could be obtained only in surface cultures [51,52]. Addition of pellet enhancers or application of very carefully regulated stirring regimes might overcome this limitation to further scalability [53]. Application of amino acids based medium supplemented with crystalline cellulose enabled submerged cultures of MF458. This condition was used for successful transfer into stirred tank reactor as the basis for upscaling of the production. To our knowledge, this is the first report on STR conditions for *T. geodes* secondary metabolite production. 

## 4. Methods

### 4.1. Isolation and Taxonomy of MF458

Strain MF458 was isolated by Dr. Karsten Schaumann from a sponge sample. DNA extraction, amplification of the internal transcribed spacer region (ITS) and the 18S rRNA gene, as well as sequencing were performed as described by [30] with slight modifications, centrifugation of the DNA at 8000× *g* and 35 cycles of DNA amplification. The DNA sequences were deposited in GenBank under the accession number KY696657, for the 18S rRNA sequence (MF458_18S), and KY696658, for the ITS region (MF458_ITS1). Cryopreserved stock cultures of strain MF458 were kept at −100 °C using the Microbank system (Microbank system, MAST DIAGNOSTIKA, Reinfeld, Germany). Closest relatives were identified by sequence comparison with the NCBI Genbank database using BLAST (Basic Local Alignment Search Tool) [54]. Sequence similarity values were determined with the “bl2seq” tool of the NCBI database [55].

### 4.2. Fermentation and Crude Extract Production

The fungal strain was cultivated in the following media: casamino acids glucose medium [56], casamino acids medium at half and double concentration, casamino acids supplemented with B12 (5 g/L), GM medium (as modified by [57]), casamino acids supplemented with molasses (80 g/L) and modified Wickerham medium [58]. Cryopreserved stock cultures were activated on WSP agar (3% NaCl) at 28 °C in the dark for 7 days. Main cultures were done in 2 L EM flasks (containing 1000 mL medium) using the different conditions. Flasks were inoculated using agar pieces from precultures (Ø = 2.6 cm) and incubated at 22 °C in the dark, at 120 rpm or using standing conditions. Crystalline cellulose (10 g/L), paper (40 g/L), gelatine (18 g/L), CaCO_3_ (15 g/L), and agar (3 g/L) were added as pellet enhancers.

For isolation of compounds, fermentation was done in multiple shake flasks containing casamino acids medium, a temperature of 22 °C and standing conditions.

The cultivation was subsequently scaled up in stirred tank reactors in 10 L (Braun Biostat MD, glass tank), containing 8 L casamino acid medium supplemented with 10 g/L crystalline cellulose (Carl Roth, Karlsruhe, Germany). Precultures were established in the same medium and cultivated for 20 days at 22 °C, 120 rpm. The inoculation volume was 100 mL (1.25%). The fermenter was run at 25 °C with pH, oxygen, and stirring speed being measured and oxygen being controlled to a minimum of 30% air saturation (flow up to 400 L/min, stirrer speed up to 200 rpm). Foam formation was stopped by addition of antifoam (Sigma, Taufkirchen, Germany). A second fermentation run was performed using the same conditions but controlling the pH at 4.3 using 3 M NaOH. 

All samples and final harvests of the fermentations were extracted using liquid-liquid extraction. 2 volumes of ethyl acetate were added to one volume of culture, homogenized and separated by means of separating funnels. The organic phase was washed using distilled water and dried *in vacuo* at 40 °C. Extracts were stored at −20 °C until use.

### 4.3. Anti-Tumour Assay-Guidance for Purification and Pure Compound Evaluation

Crude extracts and HPLC fractions were profiled at three concentrations against MCF-7, M14 and 786-0 cell lines in a 96-well microtitre assay format using the neutral red cell viability protocol to identify those containing potentially useful anticancer components [19]. The 100-fold concentrated marine fungal fermentation extracts and HPLC fractions concentrated to dryness and re-dissolved in DMSO-methanol (3:1) were tested for anti-tumour activity at three different dilutions: (A) 1/200, (B) 1/1000, and (C) 1/5000, to facilitate ranking of activities detected based on potency. This resulted, after two or three purification steps, in the isolation of single active compounds. These pure compounds were then characterized in a wider range of cell lines from the NCI60 panel. 

### 4.4. Purification and Identification of Compounds ***1**–**8***

The ethyl acetate extract of a 10 L fermentation in multiple shake flasks was dissolved in DMSO-methanol (3:1) and chromatographed in multiple injections on a Waters Xbridge phenyl column (19 × 100 mm) eluted with a linear water-acetonitrile gradient in the presence of 0.1% formic acid, increasing from 10% to 100% acetonitrile over a period of 8 min, holding at 100% acetonitrile for a further 5 min before returning to the starting composition in 1 min and re-equilibrating for another 6 min, at a flow rate of 17 mL/min. After the first minute, eluate fractions were collected every 35 s (24 fractions in total). These fractions were concentrated to dryness, redissolved in DMSO-methanol (3:1) and aliquots diluted 1/10 for assay against tumour cell lines as described above. Based on the assay results and HPLC-MS analysis of the chemical contents of the active fractions, fractions were combined and further purified as follows. The fractions (5–9) eluting between 3 and 5 min were concentrated and further purified by chromatography on a Waters Symmetry Shield RP8 column (19 × 100 mm) eluted isocratically with 25% aqueous acetonitrile containing 10 mM ammonium formate and 0.1% formic acid at a flow rate of 17 mL/min with UV detection at 352 nm. The peaks eluting between 8–10 and 11–13 min were separately collected and rotary evaporated to remove acetonitrile before applying to Mega Bond Elut Plexa solid phase extraction (SPE) columns (Agilent Technologies, Santa Clara, CA, USA, 500 mg columns) that had been pre-conditioned with methanol followed by water, for de-salting. The columns were then washed with several column volumes of water and eluted with acetonitrile, and the acetonitrile eluates concentrated to dryness to yield compounds **5** (17 mg) and **6** (24 mg).

The Xbridge phenyl column fractions (10–13) eluting between 5 and 7 min were combined and further purified by chromatography on a Symmetry Shield RP8 column (19 × 100 mm) eluted with a linear gradient increasing from 35% to 65% acetonitrile in water in the presence of 10 mM ammonium formate and 0.1% formic acid over a period of 15 min at a flow rate of 17 mL/min with UV detection at 254 nm. Eluate collected from 6 to 8 min was concentrated to dryness and purified by chromatography on the Symmetry Shield RP8 column eluted isocratically with 42% aqueous acetonitrile containing 10 mM ammonium formate and 0.1% formic acid at a flow rate of 17 mL/min with UV detection at 230 nm. The peak eluting between 5 and 6 min was collected, de-salted by SPE as above and concentrated to dryness to yield **2** (1.3 mg). The peak eluting between 8 and 11 min was collected, concentrated to dryness and purified by chromatography on the Symmetry Shield RP8 column eluted isocratically with 35% aqueous acetonitrile containing 10 mM ammonium formate and 0.1% formic acid at a flow rate of 17 mL/min with UV detection at 250 nm. The peak eluting between 21 and 23 min was collected, de-salted by SPE as above and concentrated to dryness to yield **3** (15 mg). The peak eluting between 11 and 14 min was concentrated to dryness and purified by chromatography on the Symmetry Shield RP8 column eluted isocratically with 45% aqueous acetonitrile containing 10 mM ammonium formate and 0.1% formic acid at a flow rate of 17 mL/min with UV detection at 250 nm. The peak eluting between 7 and 8 min was collected, de-salted by SPE as above and concentrated to dryness to yield **7** (9 mg). Eluate collected from 14 to 15.5 min was concentrated to dryness and purified by chromatography on the Symmetry Shield RP8 column eluted isocratically with 55% aqueous acetonitrile containing 10 mM ammonium formate and 0.1% formic acid at a flow rate of 17 mL/min with UV detection at 300 nm. The peak eluting between 12 and 13 min was collected, de-salted by SPE as above and concentrated to dryness to yield **8** (5 mg). Eluate eluting between 17 and 18 min yielded **1** (2.4 mg).

### 4.5. Assessment of Compound Cytotoxicity

Cell lines used were part of the NCI60 panel and maintained using RPMI-1640 medium (PAA, Hesse, Germany) containing 2 mM glutamine, 100 U/mL penicillin G, 100 mg/mL streptomycin, and 10% fetal calf serum (FCS). At about 80% confluence, cells were rinsed with Dulbecco’s phosphate-buffered saline (DPBS), trypsinised, suspended and counted in RPMI-1640 medium before seeding into 384-well plates. Cells were seeded at 20 µL per well in different densities (see Appendix A Table A4 for complete overview) in white, 384-well, PS, Cellstar plates (Greiner Bio-One GmbH, Frickenhausen, Germany) and incubated at 37 °C in the presence of 5% carbon dioxide. At 24 h post seeding, baseline growth was assessed using a control plate and CellTiter-Glo reagent (CTG reagent, Promega Inc., Madison, WI, USA). Briefly, 20 µL of CTG detection mix was added, and plates were analysed on an EnVision Multimode reader (PerkinElmer, Waltham, MA, USA) after 10 min incubation in the dark. In parallel assay plates were dosed with compounds in 11 point dose-response curves and analysed after 48 h incubation at 37 °C in the presence of 5% carbon dioxide using CellTiter-Glo as described. Raw data were normalized to percent of cell growth by using the baseline growth and the corresponding high control (C), containing only the solvent DMSO (Carl Roth, Karlsruhe, Germany). The measured luminescence signal of a certain sample (S) was converted into percent of cell growth compared to the average signal of the baseline control (B). In case of a sample signal higher than the average baseline the following formula was used: percent effect = (S − B)/(C − B) × 100. In case of a sample signal lower than the average baseline the following formula was used: percent effect = (S − B)/B × 100. This relative growth was used to calculate cell viability and proliferation parameters (GI50, LC50, and TGI) as described elsewhere [59]. Briefly the GI50 value corresponds to the concentration were growth is reduced to 50%, the TGI corresponds to full growth inhibition (100%) and the LC50 corresponds to 50% cell death compared to the baseline measurement. Data analysis was performed on a single plate level using ActivityBase software (IDBS Ltd, Guildford, UK). All data were recorded 3 times or more and average and estimated standard deviation of the corresponding proliferation parameters (GI50, LC50, and TGI) were calculated using Microsoft Excel (Microsoft Inc., Redmond, WA, USA). Values are given without estimated standard deviation, in case they were identical, resulting in a standard deviation of 0, or when only one of the three replicates was reaching the specific proliferation parameter. For hit compounds, a counter assay was used to determine the influence of compounds on CTG performance.

### 4.6. General

HPLC-mass spectrometry (HPLC-MS) to support compound purification and characterisation was conducted on a system comprising a 2795 Alliance HT Separations Module (Waters, Milford, MA, USA), a 2996 Photodiode Array Detector (Waters, Milford, MA, USA) an Acquity SQ detector (Waters, Wexford, Ireland) and a PL-ELS 2100Ice Evaporative Light Scattering Detector (Polymer Laboratories, Church Stretton, UK). The standard analytical HPLC method employed a SymmetryShield RP8 column (3.5 µm; 4.6 × 75 mm) eluted with a linear gradient of 10%–95% MeCN in water, containing 10 mM ammonium formate + 0.1% formic acid, at a flow rate of 1 mL/min, held at 95% MeCN for 1.0 min before returning to initial conditions over 0.5 min; total run time 12 min. Putative molecular weights, UV-Visible maxima and producing organism taxonomic data were used to search two natural products databases to identify known compounds: Antibase and the Chapman and Hall Dictionary of Natural Products. NMR spectra were recorded on a DRX500 spectrometer (500 and 125 MHz for ^1^H and ^13^C NMR, respectively, Bruker, Karlsruhe, Germany) using the signals of the residual solvent protons and the solvent carbons as internal references (δ_H_ 3.31 and δ_C_ 49.15 ppm for CH_3_OH-*d*_4_). High-resolution mass spectra were obtained on a micrOTOF II spectrometer (Bruker Daltonics, Bremen, Germany) using an ESI ion source in negative mode. UV spectra were obtained with a SpectraMax Plus 384 Microplate Spectrophotometer (Molecular Devices, Sunnyvale, CA, USA). The optical rotation was determined using an AA10 Automatic Polarimeter (Optical Activity Ltd., Huntingdon, UK). IR spectra were measured using a Spectrum 100 FTIR spectrometer (Perkin Elmer, Bridgeport, CT, USA) by diffuse reflectance using a thin film.

Analytical HPLC-UV/MS to support fermentation development was conducted on a VWR-Hitachi LaChrom Elite system (pump L-2130, diode array detector L-2450, autosampler L-2200 and column oven L-2300) (VWR, Darmstadt, Germany) with a Phenomenex Onxy Monolithic column (C18, 100 × 3.00 mm) (Phenomenex Inc., Aschaffenburg, Germany) applying a gradient of 0.1% formic acid in H_2_O (A) and 0.1% formic acid in acetonitrile (B): 0 min 5% B, 4 min 60% B, 6 min 100% B; flow 2 mL·min^−1^. Coupling of the HPLC system to a Bruker esquire4000 ESI-ion trap (Bruker Daltonics, Bremen, Germany) allowed mass detection. Samples were solved in MeOH and filtered (0.2 µm) before application to the column.

## 5. Conclusions

In summary this report shows that the marine isolate of *Tolypocladium geodes*, analysed as part of the MARINE FUNGI FP7 program, is a very effective secondary metabolite producer. It therefore shows that the investigation of new marine isolates of well-known terrestrial fungal species opens up the possibility to discover new compounds with relevant biological activities. The reported malettinin E and also the acyl tetramate tolypocladenol C were not previously described from terrestrial isolates. The assay-guided purification approach, which was used to characterise the extracts, resulted in the characterisation of molecules with potent anti-tumour effects and a predominantly anti-proliferative activity profile. The most potent activities found were due to compounds known to have anti-tumour effects, namely efrapeptin D (**2**) and pyridoxatin (**3**), but also new active molecules were discovered. The acyltetramates tolypocladenol A_1_/A_2_ (**8**) as well as the novel tolypocladenol C (**7**) only had very moderate anti-tumour potency, but the malettinins B and E (**5** and **6**) show more potent effects and are being evaluated further.

In analogy to reported biosynthesis pathways it can be assumed that the marine species of *Tolypocladium* expresses at least 5 biosynthetic pathways under conditions investigated to date. The compounds of most interest as potential anti-tumour leads were the malettinins, and production of these was significantly improved by using tailored culture conditions. The impact of media and cultivation conditions on the secondary metabolite profile increases the relevance of MF458 as a production host and shows that marine isolates, even of well-studied terrestrial species, are definitely worth exploring as serious sources for new and biologically active natural products. 

## Figures and Tables

**Table 1:** Malettinin B (**5**) yields from different culture conditions. Malettinin B was purified from the respective culture broth.

Condition	Malettinin B Yield in mg/L
Standing Erlenmeyer flask	2.28
Shaking Erlenmeyer flask	2.43
STR without pH control	18.2
STR with pH control	10.3

## Supplementary Materials

The following are available online at www.mdpi.com/1660-3397/15/4/84/s1, Supplementary Figure X1: ^1^H NMR spectrum (CD_3_OD) of tolypocladenol C (**7**), Supplementary Figure X2: ^13^C NMR spectrum (CD_3_OD) of tolypocladenol C (**7**), Supplementary Figure X3: COSY NMR spectrum (CD_3_OD) of tolypocladenol C (**7**), Supplementary Figure X4: HSQC NMR spectrum (CD_3_OD) of tolypocladenol C (**7**), Supplementary Figure X5: HMBC NMR spectrum (CD_3_OD) of tolypocladenol C (**7**).

## Appendix A

**Table A1:** Physico-chemical properties of tolypocladenol C.

Properties	Tolypocladenol C
Appearance	Pale yellow semi-solid/gum
UV (MeOH) λ_max_ (log ε)	219 (3.97), 241_sh_ (3.89), 280 (3.95) nm
ESI^+^-MS (*m*/*z*)	358 (MH^+^)
ESI^−^-MS (*m*/*z*)	356 ([M − H]^−^)
Molecular formula	C_21_H_27_NO_4_
HRESI^+^-MS (*m*/*z*)	
Found for MH^+^	358.2016
Calculated	358.2013
[α]_D_^20^	−135.2° (c 0.016, MeOH)
IR µ_max_	3250, 2963, 2920, 1654, 1603, 1516, 1451, 1378, 1336, 1269, 1238, 1172, 1105, 1018, 968, 952, 816 cm^−1^

**Table A2:** ^1^H and ^13^C NMR assignments of tolypocladenol C.

No.	*δ* ^1^H (*J* [Hz])	*δ* ^13^C
1		
2		176.7
3		103.0
4		198.2
5	3.95 dd (6.4, 3.9)	63.0
6	2.98 dd (13.9, 4.2) 2.82 dd (13.9, 6.0)	37.9
7		197.3
8	3.79 m	36.5
9	1.72 m 1.04 dd (8.8, 4.4)	41.6
10	1.29 m	32.5
11	1.95 m 1.77 m	41.5
12	5.38 m	130.9
13	5.38 m	127.0
14	1.63 m	18.1
15	0.80 d (6.5)	20.0
16	1.01 d (6.8)	18.8
1′		128.1
2′	6.99 d (8.4)	131.8
3′	6.65 d (8.4)	116.0
4′		157.2
5′	6.65 d (8.4)	116.0
6′	6.99 d (8.4)	131.8

Referenced to CD_3_OD signals at 3.31 and 49.00 ppm.

**Table A3:** Biological activities of the identified natural products in a selection of the NCI60 cancer cell line panel. Data given as µM. If compound concentration was not sufficient to reach cytostatic or cytotoxic effects, the value is given as #. If effect was not analysed in this cell line, table shows nd.

Cell Line	Origin	Value	Cyclosporin A	Efrapeptin D	Pyridoxatin	Malettinin B	Malettinin E	Tolypocladenol C	Tolypocladenols A_1_/A_2_
UO-31	Renal carcinoma	GI_50_	18 ± 0.8	6.5 ± 7.5	4.4 ± 0.5	34.5 ± 21.1	33 ± 3.6	49.1 ± 21.8	nd
		TGI	99	59 ± 56.6	9.6 ± 2.3	59	638.6 ± 143.5	214.4 ± 114.6	nd
		LC_50_	310	240	26	98	#	440	nd
786-O	Renal adenocarinoma	GI_50_	46	1.6 ± 1	3.9 ± 1.6	20.4 ± 5.3	13.1 ± 2.3	75	19.6 ± 19
		TGI	#	20 ± 21.1	10.1 ± 5.8	25	29.2 ± 10.1	#	149 ± 114.6
		LC_50_	#	325 ± 445.5	21.5 ± 14.7	#	142.5 ± 106.1	#	#
TK10	Renal carcinoma	GI_50_	17 ± 3.4	3.7 ± 4.4	12.2 ± 7	19.8 ± 5.9	6.6 ± 1.3	97.5 ± 55	nd
		TGI	49	800	74 ± 28.2	#	#	104.5 ± 58.5	nd
		LC_50_	#	#	250 ± 134.5	#	#	110.5 ± 63.1	nd
SN12C	Renal carcinoma	GI_50_	#	1.9 ± 0.4	6.3 ± 0.8	20.5 ± 10.6	16 ± 7.1	#	nd
		TGI	#	10.2 ± 4	17.3 ± 15.1	57	45	#	nd
		LC_50_	#	#	#	#	120	#	nd
UACC-62	Malignant melanoma	GI_50_	#	#	9.2 ± 1.1	38 ± 2.8	10.5 ± 0.7	58.5 ± 13.4	nd
		TGI	#	0.4 ± 0.3	22 ± 7.1	450	#	78 ± 9.9	nd
		LC_50_	#	#	51	#	#	92 ± 7.2	nd
SK-MEL-28	Malignant melanoma	GI_50_	#	1.3 ± 1.4	11 ± 11.3	#	61.5 ± 30	7.8	46
		TGI	2.9	11	62.7 ± 58.9	#	760	#	51
		LC_50_	#	#	42	#	#	#	52
M14	Amelanotic melanoma	GI_50_	12.5 ± 1	2.1 ± 1.1	2.2 ± 1.6	13.7 ± 2.5	9.6 ± 4.6	#	nd
		TGI	67 ± 24.5	39	4.1 ± 2.4	102 ± 25.5	36 ± 10.1	#	nd
		LC_50_	#	440	25 ± 5.7	370	244.5 ± 259.4	#	nd
U251	Glioblastoma	GI_50_	40 ± 24	#	4.5 ± 0.8	18.5 ± 0.7	13.5 ± 2.1	56.5 ± 6.4	nd
		TGI	#	5.6	8.2 ± 4	#	48.5 ± 30.4	64 ± 14.1	nd
		LC_50_	#	#	24.5 ± 10.6	#	#	69 ± 19.8	nd
SF-539	Gliosarcoma	GI_50_	12.5 ± 1.3	1.2 ± 0.7	5 ± 1.2	14.5 ± 2.1	9.4 ± 2.6	#	nd
		TGI	17.5 ± 0.7	61.6 ± 116.8	12.3 ± 7.5	47 ± 18.4	27.2 ± 10.9	#	nd
		LC_50_	26	101.3 ± 155.1	40 ± 1.4	#	250	#	nd
RPMI 8226	Multiple myeloma	GI_50_	7.5 ± 2.8	#	2.9 ± 0.4	49.5 ± 0.7	27.5 ± 3.5	#	19.5 ± 7.8
		TGI	12 ± 2.8	#	5.2 ± 0.6	53 ± 2.8	31 ± 7.1	#	38 ± 29.7
		LC_50_	34	0.7 ± 0.3	8.5 ± 0.8	55.5 ± 4.9	#	#	#
HL-60	Procyelo-cytic leukemia	GI_50_	14.3 ± 2.6	0.9 ± 1.3	3.1 ± 2.4	29 ± 5.2	11.4 ± 4	#	nd
		TGI	13.5 ± 0.7	2.1 ± 2.3	3.3 ± 2.5	37.3 ± 4.5	14.7 ± 3.7	#	nd
		LC_50_	14.5 ± 2.1	7.5 ± 3.1	3.7 ± 2.6	48 ± 6.6	20.4 ± 6.1	#	nd
OVCAR-5	Ovarian carcinoma	GI_50_	#	0.2 ± 0.1	4.5 ± 2.9	50 ± 1.4	20.5 ± 18.1	#	41.5 ± 16.3
		TGI	#	5.8 ± 2.9	31 ± 9.9	60.5 ± 13.4	54.7 ± 2.1	#	68
		LC_50_	#	66.5 ± 44.8	101.5 ± 26.2	69.5 ± 24.7	64 ± 11.3	#	81
OVCAR-3	Ovarian carcinoma	GI_50_	#	5.8 ± 9.7	9.6 ± 20.6	22 ± 12.5	16.4 ± 9.6	69 ± 34.2	57 ± 14.1
		TGI	#	273.3 ± 240.9	3.6 ± 2.3	25.5 ± 20.5	16.1 ± 6.9	84.7 ± 47.9	111 ± 41
		LC_50_	43	57.6 ± 97.3	28.3 ± 12.1	#	#	89 ± 52.8	175 ± 77.8
IGR-OV1	Ovarian adeno-carcinoma	GI_50_	5.3 ± 4.8	0.3 ± 0.1	3.5 ± 2.4	32.7 ± 26.5	19 ± 18.8	#	#
		TGI	145 ± 7.1	30.3 ± 33.6	11 ± 2.6	51	63 ± 23.1	#	#
		LC_50_	#	1.7 ± 1.2	47.3 ± 17.1	53.5 ± 0.7	94 ± 74.5	#	#
MDA-MB-468	Breast cancer	GI_50_	5.2 ± 1.5	#	2.4 ± 2	14.3 ± 5.1	6.1 ± 0.8	72.8 ± 38.9	nd
		TGI	10.5 ± 1.1	0.2	3.3 ± 1.6	18.8 ± 3.2	8.2 ± 1.5	96.3 ± 50.6	nd
		LC_50_	29.3 ± 12.1	330	4.8 ± 1.1	27.3 ± 4.6	11.3 ± 2.7	120.5 ± 79.8	nd
MCF-7	Breast adeno-carcinoma	GI_50_	18.3 ± 4.9	0.3	2.3 ± 1.3	13.5 ± 2.1	10.2 ± 3.8	70.5 ± 20.5	120
		TGI	#	0.8	4.8 ± 3.5	19.3 ± 6.7	11.6 ± 3.4	100 ± 28.3	140
		LC_50_	#	2.8	8.1 ± 6.9	38.3 ± 28.4	15.5 ± 2.1	150	150
HCT-15	Colon adenocarinoma	GI_50_	#	11 ± 22	2.6	28 ± 1.4	9.4 ± 10.8	42 ± 1.4	46 ± 1.4
		TGI	#	16.4 ± 28.3	5.5	32 ± 2.8	25.5 ± 0.7	49.5 ± 0.7	49.5 ± 0.7
		LC_50_	#	17 ± 29.4	12	37 ± 5.7	31 ± 7.1	52	51.5 ± 0.7
DU145	Prostate carinoma	GI_50_	11.3 ± 2.4	0.6 ± 0.8	4.1	34.4 ± 8.4	16.9 ± 9.4	87.3 ± 33.2	nd
		TGI	74	267.7 ± 229.9	18	62 ± 5.7	41.4 ± 24.6	123.4 ± 61.8	nd
		LC_50_	#	455 ± 91.9	46	86	94 ± 6.9	156.6 ± 89.9	nd

**Table A4:** Seeding cell densities used for screening in 384-well plates.

Cell Line	Seeding Cell Density	CELL LINE	Initial Cell Density
UO-31	750 cells per well	RPMI8226	1200 cells per well
786-O	750 cells per well	HL-60	2000 cells per well
TK10	1200 cells per well	OVCAR-5	500 cells per well
SN12C	1200 cells per well	OVCAR-3	2000 cells per well
UACC-62	1200 cells per well	IGR-OV1	500 cells per well
SK-MEL-28	1000 cells per well	MDA-MB-468	1200 cells per well
M14	2000 cells per well	MCF-7	2000 cells per well
U251	750 cells per well	HCT-15	400 cells per well
SF-539	1000 cells per well	DU145	1200 cells per well
