# Supplementary material for: Establishing the Secondary Metabolite Profile of the Marine Fungus: Tolypocladium geodes sp. MF458 and Subsequent Optimisation of Bioactive Secondary Metabolite Production
Source: Mar Drugs. 2017 Mar 23;15(4):84. doi: 10.3390/md15040084 (PMC5408231; doi:10.3390/md15040084)
Supplement: Supplementary file 1 [file marinedrugs-15-00084-s001.pdf]

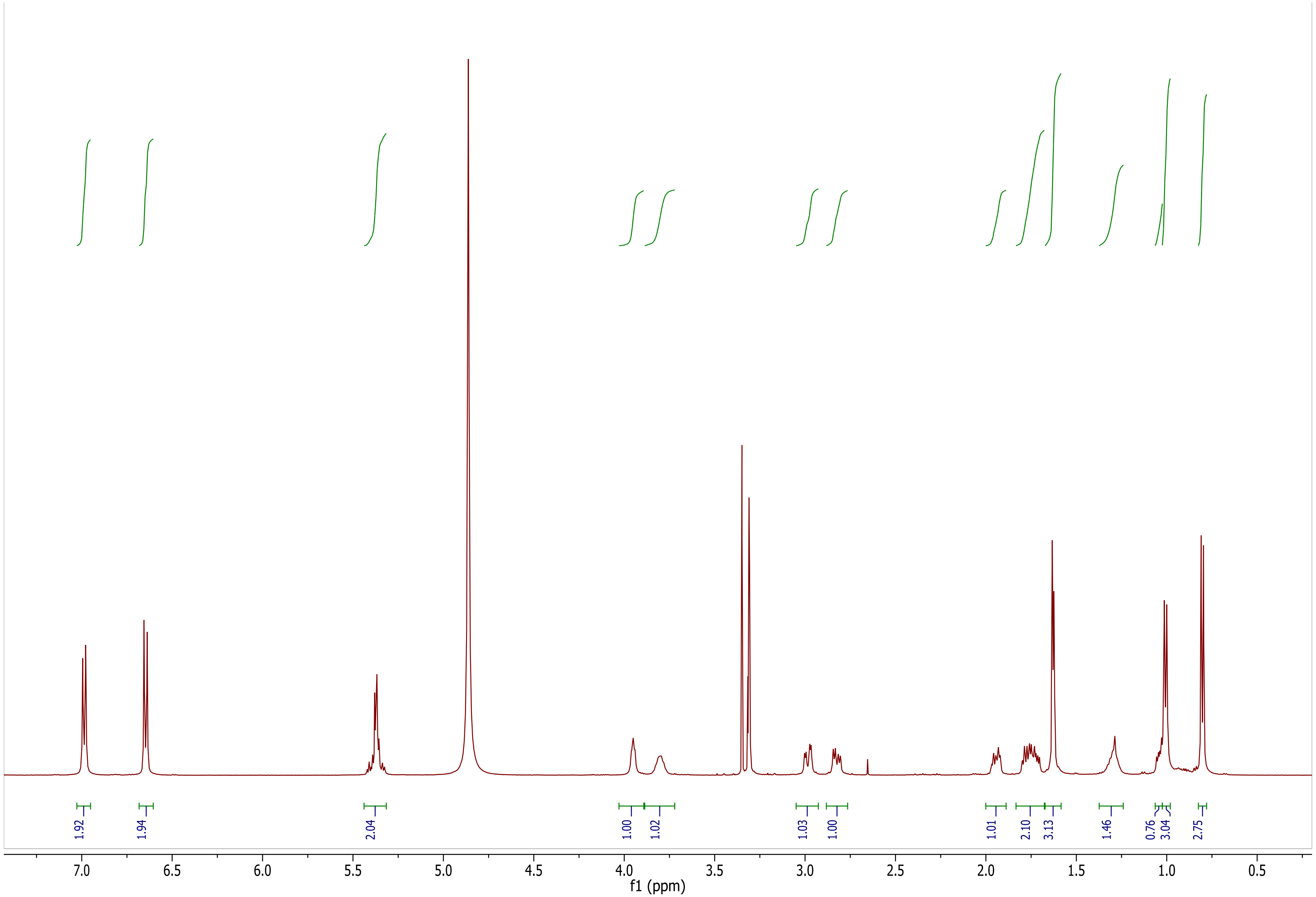

Supplementary Figure S1. ^{1}H NMR spectrum (CD_3OD) of tolypocladenol C (**7**).

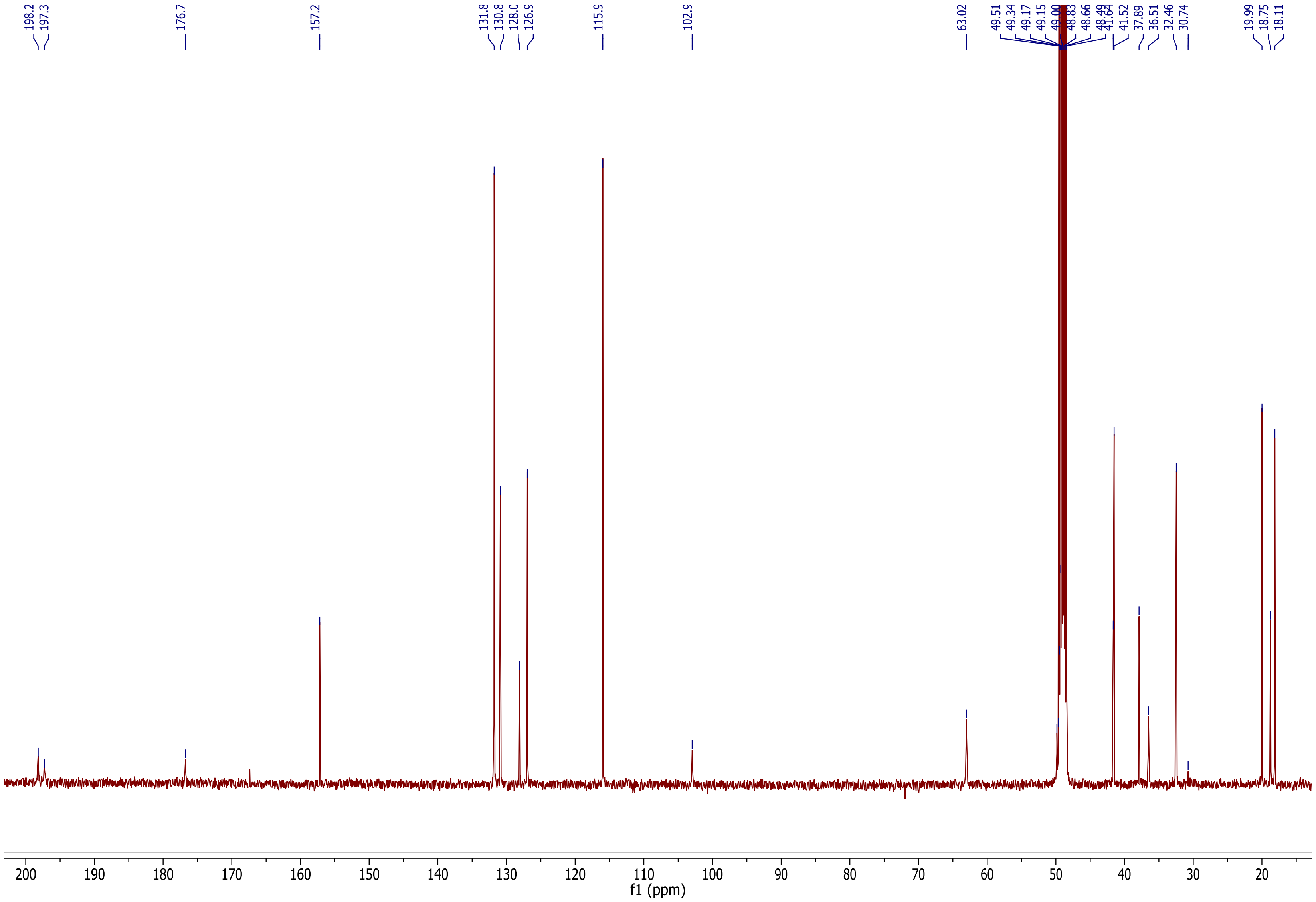

Supplementary Figure S2. ^{13}C NMR spectrum (CD_3OD) of tolypocladenol C (**7**).

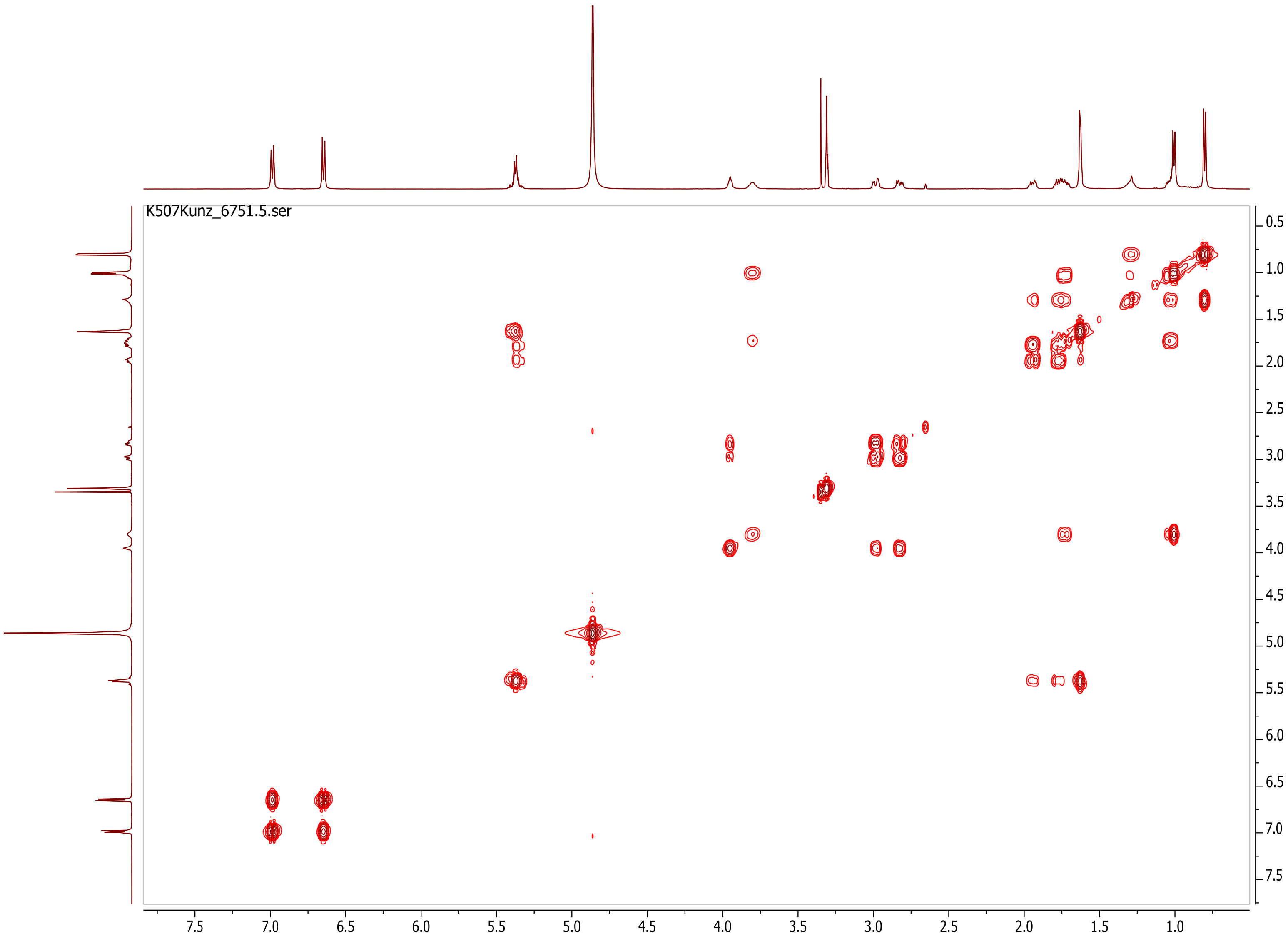

Supplementary Figure S3. COSY NMR spectrum (CD_3OD) of tolypocladenol C (**7**).

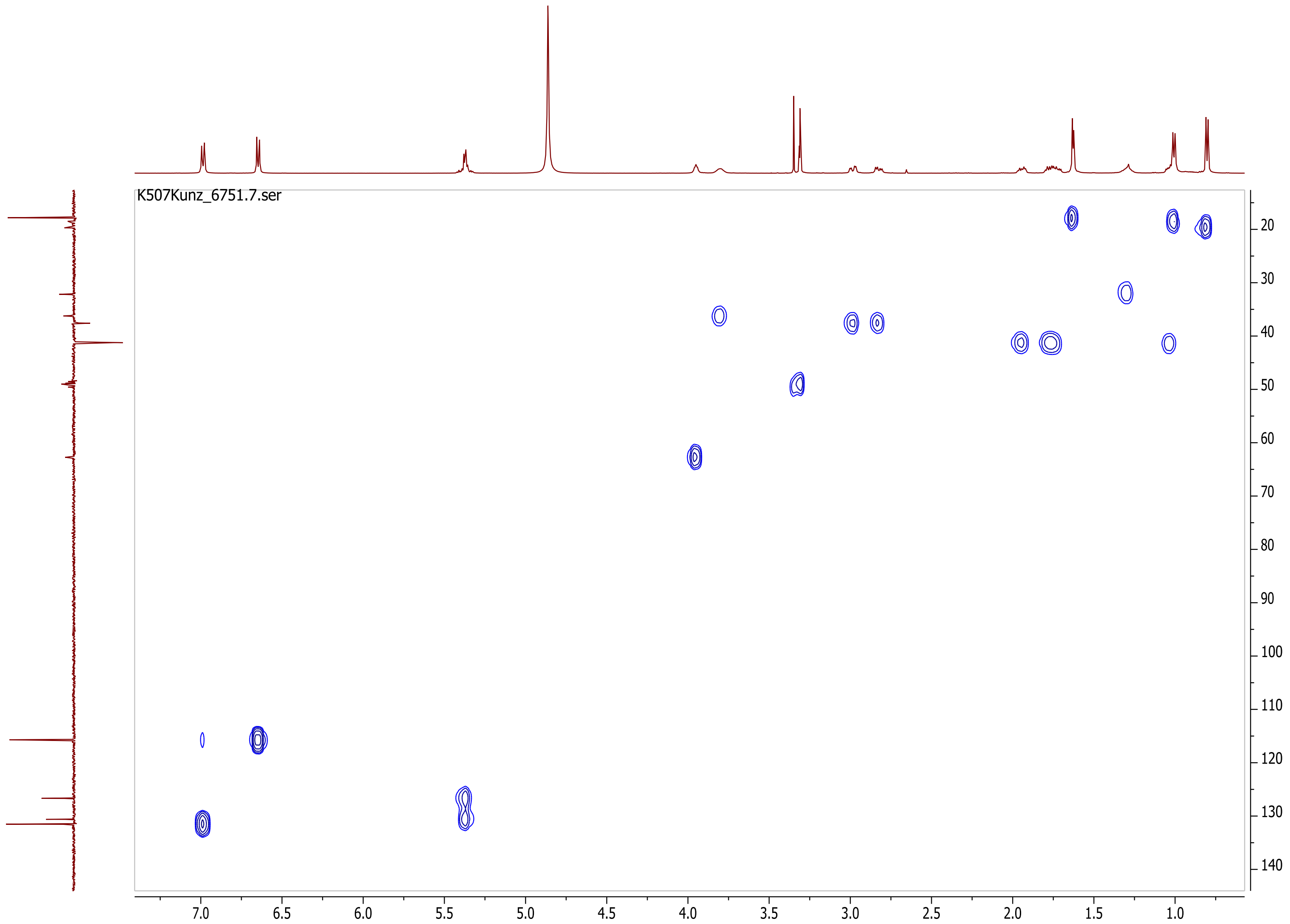

Supplementary Figure S4. HSQC NMR spectrum (CD_3OD) of tolypocladenol C (**7**).

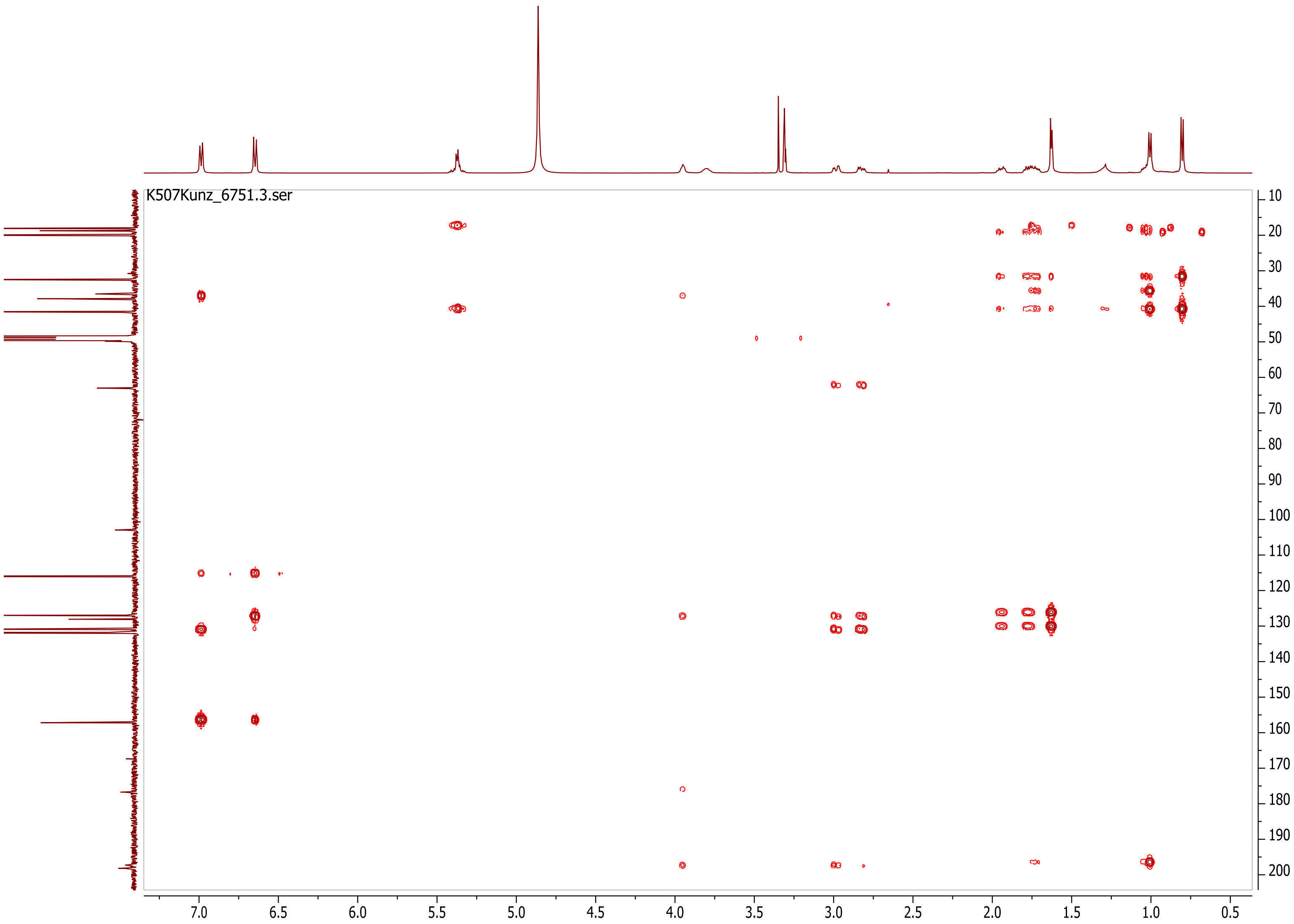

Supplementary Figure S5. HMBC NMR spectrum (CD_3OD) of tolypocladenol C (**7**).
